# Synthetic bottlebrush block copolymer prevents disease onset in Duchenne muscular dystrophy

**DOI:** 10.1073/pnas.2513599122

**Published:** 2025-10-13

**Authors:** Houda Cohen, Addeli Bez Batti Angulski, Joseph D. Quick, Taylor S. Kuebler, Brian R. Thompson, John Bauer, Dongwoo Hahn, DeWayne Townsend, Joseph F. Hassler, Benjamin J. Hackel, Timothy P. Lodge, Yuk Y. Sham, Frank S. Bates, Joseph M. Metzger

**Affiliations:** ^a^Department of Integrative Biology and Physiology, University of Minnesota Medical School, Minneapolis, MN 55455; ^b^Bioinformatics and Computational Biology Graduate Program, University of Minnesota, Minneapolis, MN 55455; ^c^Department of Chemical Engineering and Materials Science, University of Minnesota, Minneapolis, MN 55455; ^d^Department of Chemistry, University of Minnesota, Minneapolis, MN 55455

**Keywords:** Duchenne muscular dystrophy, bottlebrush block copolymer, membrane damage

## Abstract

Duchenne muscular dystrophy (DMD) is a fatal genetic disease of progressive muscle deterioration with no cure. DMD treatment requires a body-wide approach to target all diseased striated muscles: limb, respiratory, and heart. To address this, we focus studies on blocking the onset of muscle membrane instability, the primary defect in DMD, as a promising yet unmet druggable target. Here, data show the remarkable potency of a synthetic poly(ethylene oxide)/poly(propylene oxide) side chain–based bottlebrush block copolymer, ~150,000 times more potent than linear polymers, to rapidly restore contractile function to DMD skeletal muscle fibers in vitro. Strikingly, upon bottlebrush polymer delivery to DMD animals, results show highly efficacious prevention of the onset of skeletal and diaphragm muscle damage and the blocking of stress-induced cardiac injury and death in vivo. These data suggest bottlebrush polymers as a potent stand-alone muscle membrane-stabilizing therapeutic for DMD. Given DMD’s early childhood onset, together with newborn screening for DMD, bottlebrush macromolecules could be envisioned as an early therapy to preserve and protect viable muscle and potentially for other acquired or inherited diseases involving membrane damage.

## Significance

Advances in synthetic chemistry have led to an exciting class of polymers with an enormous range of molecular architectures featuring highly branched “bottlebrush” designs. A bottlebrush block copolymer (BB polymer) is identified here as a membrane stabilizer for the inherited muscle disease, Duchenne muscular dystrophy (DMD). Remarkably, this BB polymer is shown to be ~150,000 times more potent than the most effective linear triblock/diblock polymer membrane stabilizers. Furthermore, strikingly, delivery of the BB polymer in vivo is sufficient to prevent the onset of muscle damage and membrane leakiness in DMD animals. These findings reveal a connection between the branched molecular architecture in the mildly amphiphilic synthetic polymer and physiological action linked to interactions with lipid bilayers.

Duchenne muscular dystrophy (DMD) is an X-linked recessive disease of intractable muscle deterioration, with early childhood onset that is fatal in young adults due to cardiorespiratory failure (1, 2). With no cure or effective long-term treatment for DMD, great urgency centers on new therapy development. To date, multiple ongoing gene-based clinical trials for DMD have missed key functional milestones, including the termination of programs following poor clinical-trial outcomes (3–5). Collectively, this underscores the pressing need to consider alternative therapeutic approaches for DMD. In addition, the increased access to newborn screening for DMD presents new opportunities for treatments that could be applied at the very earliest stages of disease progression (1, 6). Ideally, efficacious treatment would be applied prior to the onset of marked membrane damage, denoted clinically by the release of the muscle enzyme creatine kinase into the serum that is commensurate with the loss of functional muscle tissue characteristic of the progressive nature of DMD (6). If the onset of muscle leakiness and muscle damage could be prevented, this would be expected to significantly preserve viable muscle in the long term.

To this end, multiple ongoing efforts are focused on developing DMD disease-altering drugs, including natural and synthetic small molecules (1). One compelling approach features the development of synthetic diblock and triblock copolymers designed to interface with unstable muscle membranes to protect and preserve functional muscle in DMD (7). Previously, related linear PEO–PPO–PEO triblock copolymers have proven to be safe and nonimmunogenic in humans (1). While attractive, these first-in-class linear synthetic polymers require large doses (~460 mg/kg in vivo; ~150 µM in vitro) to achieve DMD therapeutic efficacy, thus hindering further development (7, 8).

More recently, synthetic bottlebrush polymers have emerged as a unique type of branched polymer featuring architectures of densely grafted side chains on a backbone (9, 10). Compared to linear polymer structures, the bottlebrush polymer architectural space is massive (10, 11). Bottlebrush macromolecules can be designed in myriad conformations involving the assembly and dynamic control of side-chain polymer branches upon a defined chemical backbone, wherein side-chain polymerization length, density, and composition along the backbone confer unique physicochemical properties, including overall macromolecule shape and stiffness. In the present work, a synthetic polymer-based amphiphile, consisting of polymerized poly(ethylene oxide) (PEO) and poly(propylene oxide) (PPO) macromonomers forming a bottlebrush (BB) diblock copolymer, demonstrates proof-of-concept in preserving muscle membrane integrity in DMD (7, 12–16).

## Results

**Fig. 1.**
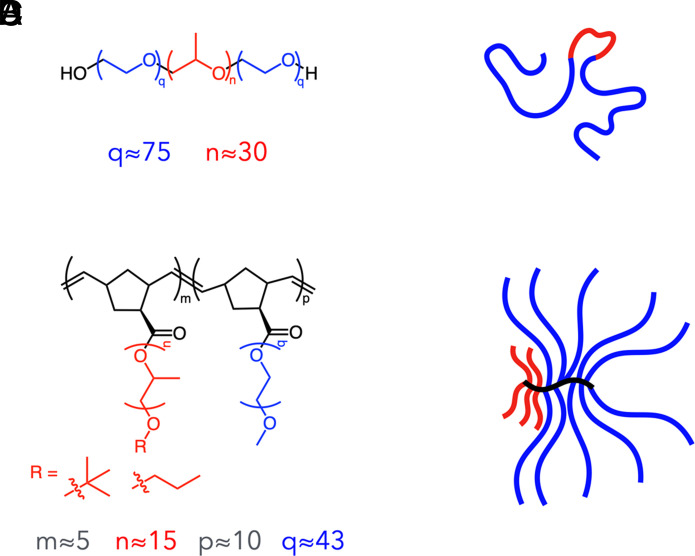
Synthetic polymer design. (*A*) Linear polymer PEO_75_–PPO_30_–PEO_75_ (P188) has two blocks of poly(ethylene oxide) (blue) flanking a core block of poly(propylene oxide) (red). The number-average degrees of polymerization for each block (*q; n*), are denoted. (*B*) Schematic representation of the linear polymer P188. (*C*) Bottlebrush polymer $B-{PEO}_{10}^{43}-{PPO}_{5}^{15}$, BB polymer herein, has side chains of poly(propylene oxide) (red) and poly(ethylene oxide) (blue) grafted onto a norbornene backbone (black). The number-average degrees of polymerization for side chains and each block (*m, n, p, q*), are denoted. Side chain density is set by each norbornene-based monomer. (*D*) Schematic representation of the BB polymer. *B* and *D* are drawn to a similar scale.

Informed by our two decades-long experience with membrane-interfacing molecules for DMD muscle, together with new advances in bottlebrush macromolecular design and synthesis, we developed bottlebrush polymer ${B-PEO}_{10}^{43}-{PPO}_{5}^{15}$ (BB polymer; Fig. 1) (9), as a candidate membrane stabilizer for testing in DMD mice. The sub- and superscripts denote the degrees of polymerization of the corresponding backbone and side chains, respectively. This BB polymer consists of two discrete blocks of PEO and PPO side chains at a w/w ratio of ~81:19 (respectively, excluding the polynorbornene backbone) and an overall average molecular weight of 26 kDa (Fig. 1).

**Fig. 2.**
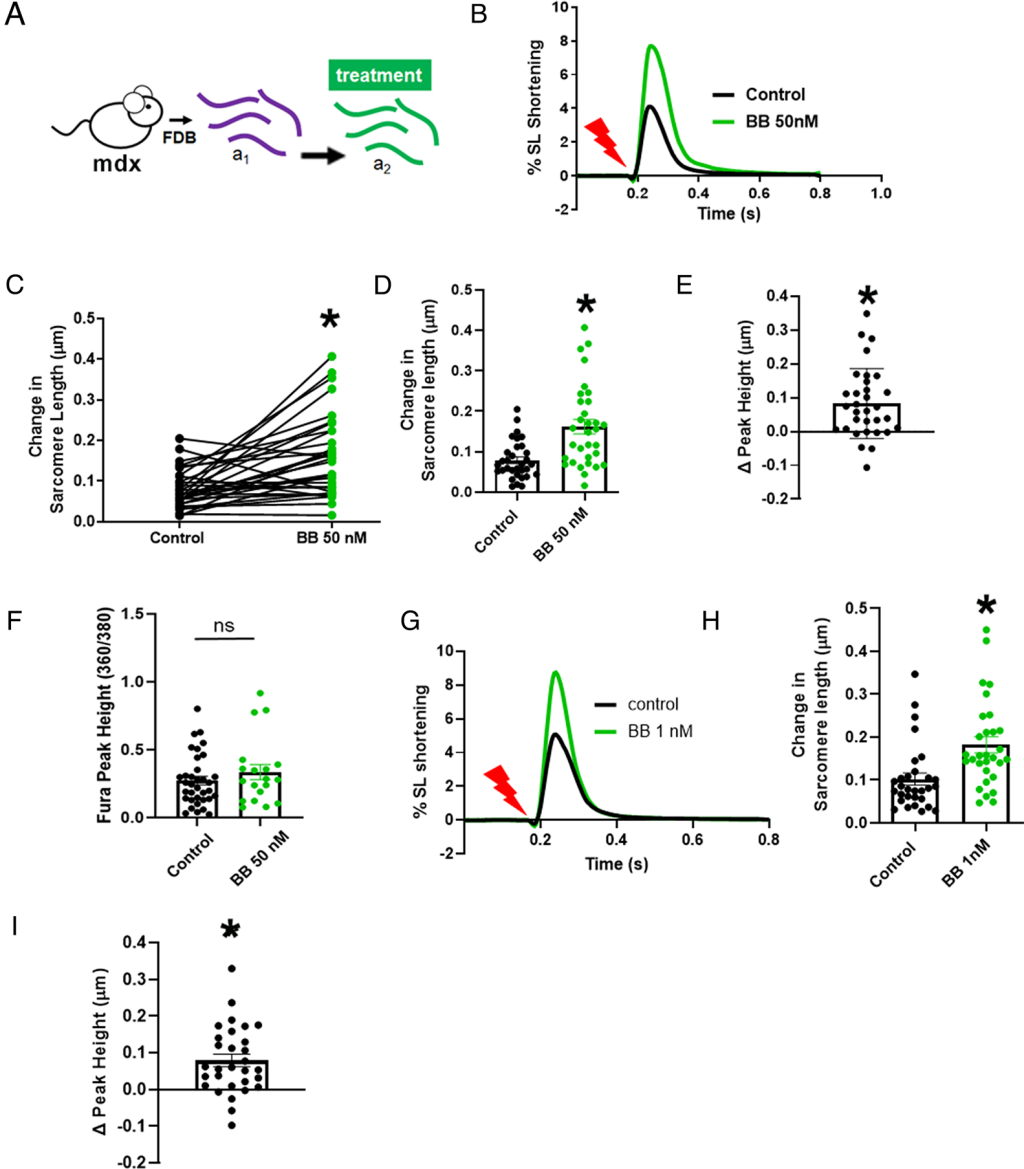
Bottlebrush polymer increases the twitch contraction amplitude of live flexor digitorum brevis (FDB) single skeletal muscle fibers isolated from dystrophin-deficient mdx mice. (*A*) Protocol. Treatments are media control and bottlebrush polymer (BB) studied longitudinally in each fiber pre (a_1_) and same fiber post (a_2_) treatment via a pairwise experimental design. (*B*) Ensemble recordings of twitch contractions before (black) and after treatment with BB polymer (BB, green trace), 50 nM. The red arrow denotes electrical pulse with data collected at 0.2 Hz. SL, sarcomere length. (*C*) Peak amplitude change in sarcomere length for paired FDBs, with each single FDB connected by lines pre (black dot) and post 50 nM BB polymer (green dots). *P < 0.0001, N = 32 pairs, paired *t* test. (*D*) Same data as *C* shown as bar graphs, mean ± SEM, *P < 0.0001. (*E*) The change in peak height from 50 nM BB minus pretreatment for every myofibers, mean ± SEM, *P < 0.0001, one-sample *t* test versus zero. (*F*) No significant difference in calcium transient amplitude, with and without 50 nM BB, mean ± SEM *P* = 0.303 unpaired *t* test, control N = 35; BB N = 19. (*G*) Sarcomere length percent shortening traces showing increased SL amplitude with 1 nM BB (green trace) compared to untreated control (black trace). Stimulation denoted by the red arrow, at 0.2 Hz pacing. (*H*) Peak amplitude change in sarcomere length for pre (black dots) and post 1 nM BB (green dots), mean ± SEM, *P < 0.0001, N = 30 pairs with the paired *t* test. (*I*) The change in peak height between control and 1 nM BB in every FDB fiber, mean ± SEM, *P < 0.0001 with one-sample *t* test (versus 0). Three independent FDB preps were performed for each group.

We first tested this BB polymer at the level of intact single skeletal muscle fibers isolated from dystrophin-deficient mdx mice, a well-validated animal model of DMD (1). Here, we used our recently established single skeletal muscle fiber flexor digitorum brevis (FDB) assay that demonstrates marked deficits in contractile performance in mdx versus control C57BL/10 FDB muscle fibers, wherein twitch peak sarcomere length (SL) shortening of mdx FDB fibers is depressed by ~70% compared to the dystrophin-replete control muscle fibers (17). Upon bathing the mdx FDB fibers in media containing the BB polymer, there was instantaneously (as fast as could be measured) a correction in the twitch contractile deficits in mdx FDB fibers (Fig. 2). In pilot studies, high potency effects of the BB polymer became evident, and detailed studies then showed FDB contractile amplitude restoration in the sub-µM range. Specifically, in pairwise measurements, FDB twitch amplitude data showed a statistically significant increase at extremely low dosing, 50 nM (Fig. 2 *A*–*E*). Here, the calcium transient amplitude during twitch contractions was not altered by the BB polymer (Fig. 2*F*). The basis of the increased twitch contraction by BB polymers, in the absence of increased Ca^2+^ amplitude, is not fully known; however, restoration in physical features of the membrane such as viscosity, stiffness, and tension could impact the contractile amplitude of the FDB fibers (17). Noting that membrane lesions have been reported in DMD muscle (18), we hypothesize that the BB polymer mechanism of action is to directly interface with and stabilize the mdx muscle membrane. Our data suggest this mechanism is independent of an effect on intracellular calcium handling. We next observed that further dilution of the BB polymer to 1 nM significantly increased FDB SL contraction amplitude (Fig. 2 *G*–*I*). This effect on twitch amplitude was lost upon BB polymer dilution to 100 pM (*SI Appendix*, Fig. S1). For comparison, we recently demonstrated, using this same experimental system, that optimized linear PEO–PPO–PEO triblock and PEO–PPO diblock polymers require ~150 µM to achieve similar improvement to mdx FDB fibers, with no effect in control dystrophin-replete FDB fibers, in vitro (17). Thus, remarkably, this BB polymer is ~150,000 times more potent than the most effective linear triblock/diblock polymer membrane stabilizers when tested at the level of the mdx live single skeletal muscle fiber. In follow-up studies, it will be important to further expand upon these findings to other muscle tests, including tetanic contractions and lengthening injury assays.

**Fig. 3.**
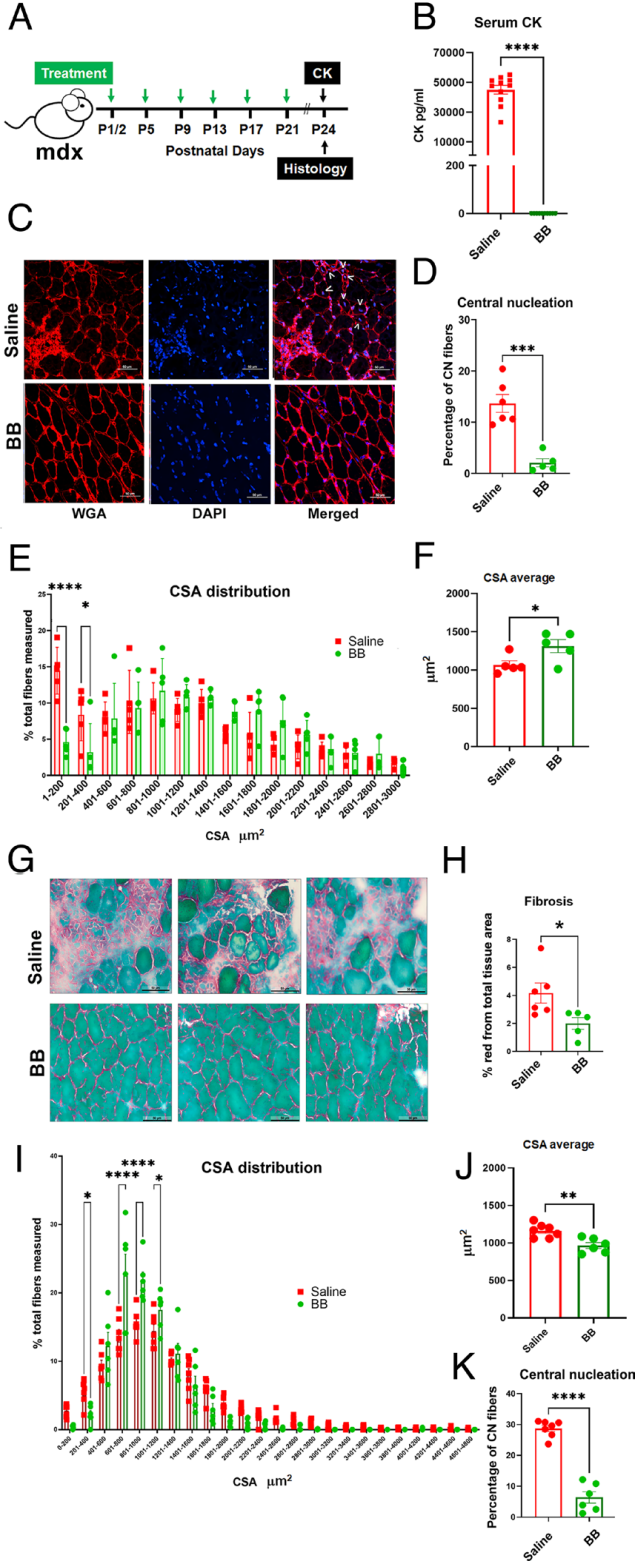
Early systemic administration of BB polymer prevents muscle membrane damage, central nucleation, and necrosis onset in dystrophin-deficient skeletal muscles and diaphragm in vivo. (*A*) Experimental timeline. Bottlebrush polymer treatment (green arrows) was administered subcutaneously on postnatal day 1 or 2 (P1/2), P5, and then every 96 h up to P21. (*B*) Serum creatine kinase (CK) at P24. N = 10 to 11 mice per group. (*C*) Representative images of tibialis anterior muscle sections stained with wheat germ agglutinin (red) and DAPI (blue) used to evaluate central nucleation (denoted by white chevrons in the saline panel, upper right), as quantified in (*D*), and fibers cross-sectional area (CSA) distribution represented in (*E*). (*F*) Tibialis anterior muscle CSA average at P24. N = 5 to 6 mice per group. (*G*) Representative fibrotic lesions detected in the saline-treated mdx diaphragm at P24, as stained with Sirius Red Fast Green. These lesions were not observed in the BB polymer–treated mdx diaphragm sections. Total fibrosis across the entire diaphragm is quantified in *H*, showing significantly more fibrosis in saline versus BB polymer–treated mdx diaphragm. N = 5 to 6 mice per group. (*I*) Diaphragm muscle CSA distribution at P24. (*J*) Diaphragm CSA average at P24. (*K*) Percentage of centrally nucleated fibers in P24 diaphragm from bottlebrush-treated and saline-treated mdx mice. N = 6 to 7 mice per group. Data are presented as mean ± SEM. Statistical testing was calculated using the two-tailed Student *t* test, *P < 0.05; **P < 0.01; ***P < 0.001, and ****P < 0.0001.

Next, we tested the bottlebrush polymer in vivo. We first sought to determine whether implementing BB polymer treatment early in disease progression, specifically prior to the well-characterized onset of muscle membrane leakage of creatine kinase (CK) into the serum and the onset of the fulminant necrotic phase of DMD in mdx mice (~17 to 20 d postnatal) (1), could be effective in altering skeletal muscle disease progression in vivo. Male mdx mice, starting at postnatal day 1 or 2 after birth (P1 or P2), were administered 0.15 mg/kg BB polymer subcutaneously, and this was continued every 4 d (P5, P9, P13, P17, and P21; Fig. 3*A*). Parameters for in vivo dosing and timing of delivery were informed by our results on isolated FDB fibers along with our recent in vivo tracer studies on PEO/PPO block copolymers (19). Animals were then euthanized at P24 according to the approved procedure by the University of Minnesota Institutional Animal Care and Use Committee (IACUC), as this period extends well over the initiation of CK release and onset of necrotic disease in mdx mice (1, 20, 21). In fact, in both mice and humans, CK release reaches a peak early in disease and then decreases over time due to the progressive loss of viable muscle tissue. Thus, as expected, serum CK was massively increased in saline-treated mdx controls at P24 (Fig. 3*B*). Most strikingly, increases in serum CK were fully prevented by the BB polymer treatment regimen (Fig. 3*B*). Because serum CK is an excellent biomarker of damage reflecting all affected DMD striated muscles, our findings of no increase in serum CK above baseline control is evidence of BB polymer efficacy protecting all striated muscles in the mdx mice. We further hypothesize that other biomarkers of muscle damage, e.g., LDH and IgG, would also be blocked by BB polymer treatment, as shown here for CK. Moreover, skeletal muscle (tibialis anterior, TA) central nucleation and TA muscle fiber cross-sectional area (CSA), which were markedly affected in saline-treated mdx muscle, as consonant with the marked cycles of necrosis/degeneration/regeneration characteristic of DMD (1, 22), were also prevented in BB polymer–treated mdx mice (Fig. 3 *C*–*F*). Then, in a separate study using the same treatment protocol as in Fig. 3*A*, experiments focused on the diaphragm muscle. In the mdx mouse, as in humans, the diaphragm muscle is damaged early in disease progression, with marked muscle necrosis and fibrosis well documented (1, 23). Notably, BB polymer treatment prevented diaphragm muscle damage as evidenced by blocking central nucleation, muscle fiber cross-section area distribution shifts, and muscle fibrosis (Fig. 3 *G*–*K*). In terms of CSA, there were differences in the fiber size distribution profiles between TA and diaphragm. These differences are attributed, in part, to the differing physiological roles of limb versus respiratory muscles. These differences notwithstanding, data show a common finding in both, notably that for the smallest fibers (CSA<400 µm^2^), there were significantly more fibers in the saline treated versus BB polymer treated for both TA (Fig. 3*E*) and diaphragm (Fig. 3*I*). This is consistent with BB polymer preserving viable muscle and blunting the degeneration/regeneration process and the formation of small CSA regenerating myofibers characteristic of DMD. Further, whereas fibrosis was significantly evident in the mdx diaphragm, based on past work we would predict even greater fibrotic lesions as the animals age. In follow-up studies, it will therefore be of interest to extend BB polymer treatment to older animals.

In parallel studies, we tested for possible toxicity using the same BB polymer treatment protocol outlined in Fig. 3*A*. A panel of kidney [blood urea nitrogen (BUN) and creatinine] and liver function biomarkers [alkaline phosphatase (ALP); aspartate transaminase (AST); and alanine transaminase (ALT)] showed no changes upon treatment, supporting a favorable safety profile for BB polymer treatment in vivo (*SI Appendix*, Fig. S2). Whereas optimization of delivery, dosing, and timing will require further experimentation, these findings are evidence that BB polymer is highly potent, safe, and effective in preserving and protecting dystrophin-deficient limb and respiratory muscles in vivo.

**Fig. 4.**
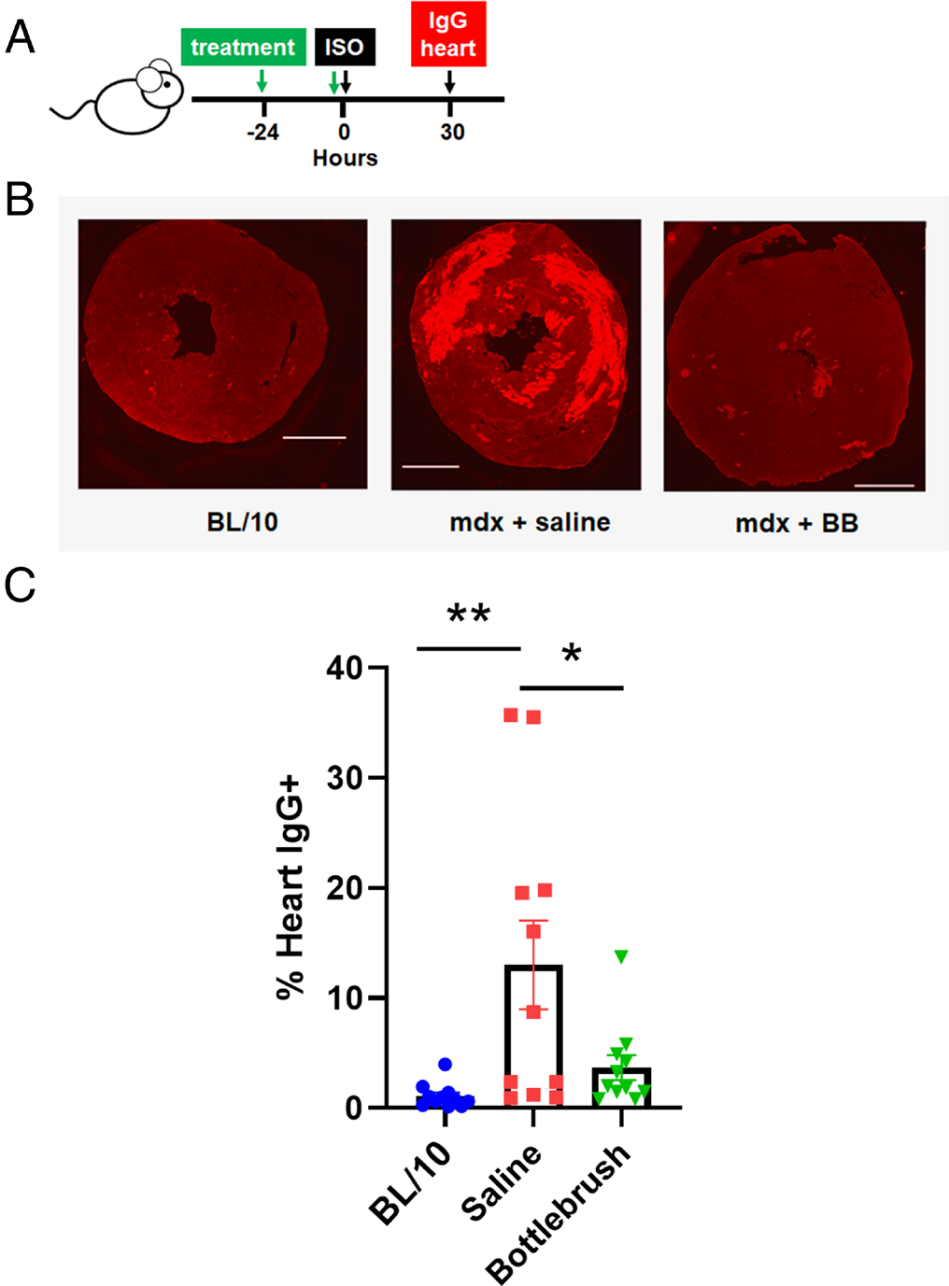
Protective effects of bottlebrush polymer on the heart during acute cardiac stress testing in dystrophin-deficient mice in vivo. (*A*) Study protocol. Treatment (BB or saline volume control) timing is shown by green arrows. Single isoproterenol (Iso) bolus cardiac stress testing at 0 h. Hearts isolated at 30 h post-Iso. (*B*) Representative whole heart transverse sections following IgG immunolabeling. (Scale bar, 1,500 µm.) (*C*) Summary graph of the % IgG positive labeling of whole heart transverse section. N = 11 samples per group. Data are presented as mean ± SEM. Statistical testing was calculated using one-way ANOVA followed by Tukey’s post hoc *P < 0.05; **P < 0.01.

Next, we tested the effectiveness of the BB polymer to prevent cardiac injury in mdx mice. This is relevant as heart disease is the main cause of mortality in DMD (1). Whereas the natural evolution of cardiac disease in mdx mice and humans can take more time to manifest (1), it is well documented that acute cardiac stress testing via isoproterenol (Iso) injection can readily unmask the deleterious effects of dystrophin deficiency on the mdx heart (8). Here, the resulting high cardiac workload induced by Iso infusion is sufficient to cause marked membrane damage in cardiac muscle leading to heart pump failure (24). Accordingly, adult mdx mice (average age, 3.5 mo) were pretreated with either BB polymer (0.15 mg/kg), a linear polymer with 80% PEO and 20% PPO (P188; 460 mg/kg), or saline vehicle control at day-1 (24 h prior to Iso injection), and at day 0, followed by a single intraperitoneal injection of Iso at 10 mg/kg (Fig. 4*A*). Hearts were then harvested and tested for immunofluorescence detection of intracellular IgG content at 30 h post–Iso injection, where the maximum Iso-induced cardiac muscle histological damage has been established (24). In healthy, dystrophin-replete myocardium, large molecules such as IgG are prevented from entry into the myocytes via stable muscle membrane barrier function (Fig. 4 *B* and *C*). Data show that the BB polymer is highly effective at protecting the dystrophin-deficient heart during acute cardiac stress testing, as revealed by blocking IgG entry into cardiac muscle in vivo (Fig. 4 *B* and *C*). However, in this cardiac stress-test assay, treatment with the linear polymer P188 (460 mg/kg) did not provide significant protection to mdx mice from Iso-induced cardiac injury (*SI Appendix*, Table S1). Moreover, Iso infusion to C57BL/10 J controls did not produce significant cardiac injury (*SI Appendix*, Table S1). In this assay, the hearts of BB polymer–treated mdx mice did not differ from control dystrophin replete hearts upon cardiac stress testing in vivo.

**Fig. 5.**
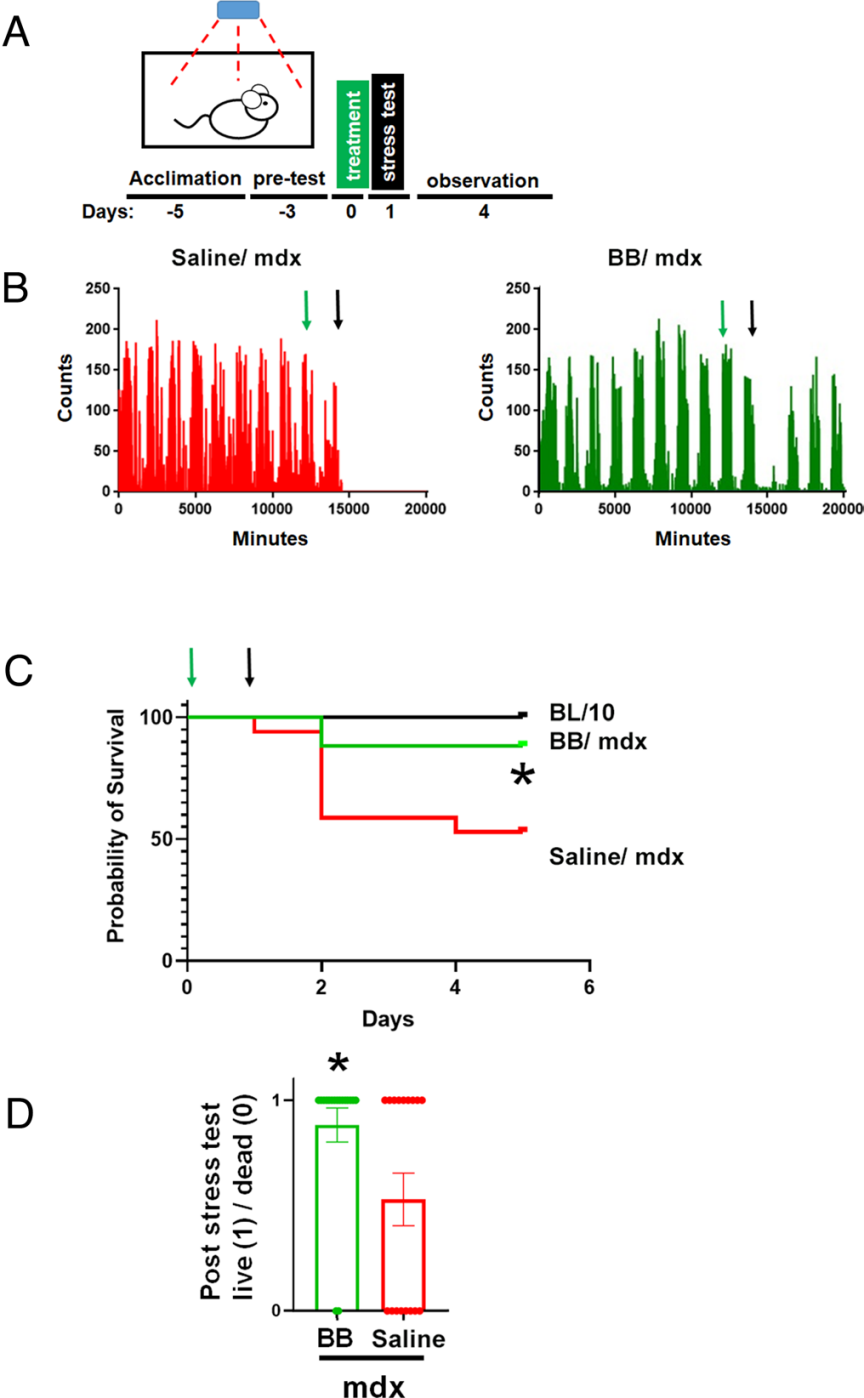
Bottlebrush polymer confers survival protection during stress testing in unrestrained dystrophin-deficient mice in vivo. (*A*) Protocol overview. Single-caged adult mdx mice were acclimated for 5 d to a noninvasive infrared-based motion-detection system (blue box /red arrows). Pretest motion data were collected for 3 d, followed by treatment (day 0) and then the Iso stress test (day 1), with animals monitored during the ensuing observation period (4 d). (*B*) Representative daily beam-break mouse activity counts for a control mdx (saline; red) and mdx + bottlebrush polymer (green). The green arrow denotes treatment, and the black arrow denotes the stress test. (*C*) Kaplan–Meier survival plots. The green arrow denotes treatment, and the black arrow denotes the stress test. * *P* < 0.05, N = 17 each group. (*D*) The fraction of animals that survived (live = 1/dead = 0), bars are mean ± SEM. Data show significant survival protection in BB versus saline mdx groups. *P < 0.05; BB (N =17; 2/17 dead) improves activity/survival over saline (N = 17; 8/17 dead).

Finally, we assessed BB polymer in preserving survival during stress testing in adult mdx animals in vivo. Live mdx mouse stress testing is invaluable in assessing the overall physiological relevance of experimental treatments as potential therapy for DMD (1). Here, in unrestrained adult mdx mice (average age, 6.5 mo) locomotor activity was documented, as done previously (25). An automated system with passive infrared sensors was positioned on the top of each cage for continuous noninvasive monitoring of mouse locomotor activity (Fig. 5*A*). For stress in mdx mice, we used a combination of handling stress (25), together with Iso stress, shown by us, and others, to markedly affect the viability of mdx mice in vivo (8, 25–27). As summarized in Fig. 5, stress unveiled a striking mortality in mdx mice wherein ~50% of the mdx mice died following stress testing while all the control C57BL/10 animals survived. Notably, pretreatment of mdx mice with BB polymer (0.15 mg/kg) conferred marked protection as evidenced by significantly increasing animal survival poststress, as compared to saline-treated mdx in vivo (Fig. 5 *B*–*D*). In contrast, pretreatment with linear block copolymer P188, at 3,000 times higher dosing (460 mg/kg), did not confer statistically significant protection to mdx mice in this stress test (*SI Appendix*, Fig. S3).

## Discussion

This work advances a synthetic bottlebrush block copolymer, ${B-PEO}_{10}^{43}-{PPO}_{5}^{15}$_,_ as a stand-alone therapeutic to stabilize damaged muscle membranes and prevent the clinical onset of disease in the mdx model of DMD. With remarkable potency, ~150,000 times more potent than previous linear triblock/diblock copolymers, ${B-PEO}_{10}^{43}-{PPO}_{5}^{15}$ restores contractile function to dystrophin-deficient skeletal muscle fibers. In addition, data show this bottlebrush macromolecule is sufficient to prevent the onset of membrane leakiness and necrotic disease in vivo. Collectively, these findings provide evidence of a highly potent and efficacious candidate DMD therapy, either as a stand-alone therapy or potentially as a combinatorial drug approach involving small molecules and/or gene/cell-based therapies.

Mechanistically, we recently investigated the interaction of the BB polymer with aqueous dispersions of liposomes, demonstrating a higher affinity of this branched macromolecule toward lipid bilayers when compared to the linear P188 triblock polymer (11). In another study, we showed that bottlebrush copolymers adsorbed on the lipid vesicle surface did not diffuse through the lipid bilayer, whereas P188 was transported to the interior cavity of liposomes, at 37 °C (28). These results suggest that the massively different action of BB polymer $B-{PEO}_{10}^{43}-{PPO}_{5}^{15}$ versus the linear P188 documented here is associated with qualitatively different mechanisms of interaction between the macromolecules and cell membranes.

To facilitate the interpretation of our findings, we next developed an all-atom model of the membrane bilayer with the inserted BB polymer. We conducted molecular dynamics-based investigations of the docking configuration of the BB polymer on the membrane bilayer to interrogate its mechanism of interaction with the lipid bilayer (*SI Appendix*, Fig. S4). Molecular dynamics simulations showed a highly stable conformation was obtained wherein the PPO-based block of BB polymer inserted into the outer leaflet of the membrane bilayer and the PEO block oriented vertically, adopting a stable “flagpole” orientation via PPO sidechains arborizing and interacting with fatty acid tails of the phospholipids (*SI Appendix*, Fig. S4). In contrast, PEO block insertion, or horizontal placement of the BB polymer relative to the membrane surface, was highly unstable (*SI Appendix*, Fig. S4). This effect was quantified with respect to localizing in tight proximity with membrane phospho-head groups of the bilayer outer leaflet (*SI Appendix*, Fig. S4*D*). In our working model, this stabilization of the membrane is proposed to arise from physically plugging the membrane lesions (delta lesions) that have been observed in DMD muscle (18).

Calculating from the FDB studies above (Fig. 2), there are approximately 2.4 × 10^9^ bottlebrush molecules per each single FDB fiber [50 FBDs plated in 200 µL of a 1 nM solution containing 1.2 × 10^11^ BB polymers]. Estimating individual BB polymer surface area of ~212 nm^2^ (BB polymer dimensions: h ~11 nm; r ~ 2.5 nm from *SI Appendix*, Fig. S3) and the exposed surface area of the FDB at ~700 µm^2^ [cylindrical geometry (not accounting for t-tubule area), FDB L = ~400 µm, radius = ~ 15 µm (17)], this translates to a total BB polymer surface area that is ~700 times greater than the total FDB membrane surface area. It has been well established that dystrophin-deficient muscle is highly susceptible to membrane damage, and this is observed ultrastructurally in the form of membrane microtear damage termed delta lesions (18). Thus, these calculations, MD simulations (*SI Appendix*, Fig. S4), and the potent cellular protective effects of the BB polymer (Fig. 2), are consistent with a mechanism of action model wherein BB polymers interface with areas of damage in muscle membranes functioning as muscle membrane stabilizers for dystrophic muscle (18).

Collectively, these data provide evidence of a highly potent, safe, and efficacious synthetic membrane-stabilizing therapeutic macromolecule for preserving and protecting dystrophin-deficient muscles in vivo. From the high potency of the $B-{PEO}_{10}^{43}-{PPO}_{5}^{15}$ polymer, we can hypothesize that an increased BB polymer–membrane interaction dwell time would markedly reduce the concentration of polymer and enable increased time required between dosing for in vivo applications. Whereas a full assessment of the optimal dosing, timing and duration of action effects, absorption, distribution, metabolism, and excretion (ADME) and safety awaits future studies, results here demonstrate a class of synthetic chemistry, bottlebrush polymers (9, 10, 29–32), with unique and remarkable chemical features making them highly attractive candidates as potent membrane stabilizers in DMD. It has been established that synthetic PEO/PPO chemical-based membrane interfacing polymers have excellent ADME and safety profiles, per long-term clinical trials in humans for other applications [Mast Therapeutics Sickle cell trials (33–39)]. This is consonant with the evidence of nontoxicity shown here with bottlebrush polymer.

Our findings gain further relevance in the context of the increased access for newborn screening in DMD (6). Thus, an opportunity arises wherein fast-acting, highly efficacious membrane-stabilizing BB polymers could be envisaged as therapy starting in the earliest days of newborn life and would be applicable to all patients regardless of specific DMD mutation. When applied early in disease, the rapid protection conferred by BB polymer is important to consider in comparison to other treatments, such as corticosteroids, that are contraindicated in DMD patients before two years of age, a time when membrane leakage is massive and muscle damage is already evident (1).

While not a cure, we speculate that early childhood administration of bottlebrush macromolecules to DMD patients could have the potential for effective life-long benefit. Given the high potency of BB polymers [~150,000 times increased potency over linear block copolymers in single muscle fibers] therapeutic levels may be obtained by small volume subcutaneous injections, analogous to the treatment regime for Type I diabetic patients who can lead a full life when taking subcutaneous insulin injections. Other routes of BB polymer delivery may also be efficacious, including inhalation (40). In other treatment scenarios, one may envisage bottlebrush polymers preserving viable muscle early on in the disease progression to then facilitate, in combination therapy, improved outcomes for gene-based treatments, for example (1). Presently, the eight FDA-approved drugs for DMD, ranging from exon skipping, gene addition, histone deacetylase inhibitors, and corticosteroids, are designed in an attempt to manage disease symptoms and improve muscle function (1). In the absence of complete full-length dystrophin gene restitution in DMD, bottlebrush polymers represent a unique stand-alone chemical-based membrane stabilizer surrogate. Thus, if muscle membrane instability and muscle tissue necrosis could be prevented in the long term by synthetic polymers, the preserved form and function of skeletal muscle, diaphragm, and heart would then be expected to markedly enhance the health and lifespan of DMD patients. We further speculate that BB polymers may also provide efficacy in acquired diseases of membrane damage, such as in ischemia/ reperfusion injury (12).

## Methods

### Animals.

All mice studies were conducted under protocols approved by the University of Minnesota Institutional Animal Care and Use Committee (IACUC) as commanded by the Federal Animal Welfare Act, NIH (NIH) guidelines, and standards of the Association for Assessment and Accreditation of Laboratory Animal Care (AAALAC International). Adult male mdx mice (C57BL/10ScSn-DMDmdx) and wild-type BL/10 mice (C57BL/10ScSn) were obtained from Jackson Labs (Bar Harbor, ME) and housed locally. The mdx strain lacks dystrophin protein and provides a well-studied model of DMD (1). Males were used as DMD is an X-linked recessive disease (1).

### Synthetic Polymers.

Bottlebrush polymer (${B-PEO}_{10}^{43}-{PPO}_{5}^{15}$; BB polymer) synthesis was performed as previously described (9). Briefly, poly(propylene oxide) (PPO) containing hydroxyl ω-terminal groups was synthesized by anionic polymerization (to *Mn* = 1,090 g/mol), resulting in 93% *tert*-butyl and 7% propylene α-end groups, the latter arising from chain transfer to monomer. Alkene chain-end impurities were removed by hydrogenation. Condensation to generate norbornene-functionalized macromonomers was achieved with 1 eq of polymer (0.05 M) and 1.5 eq of *exo*-5-norbornene-2-carboxylic acid, along with 0.2 eq of 4-dimethylaminopyridine and 1.5 eq of N,N’-diisopropylcarbodiimide in anhydrous dichloromethane at room temperature for 2 d (PEO) or 7 d (PPO). Norbornene functionality was quantified via matrix-assisted laser desorption/ionization (MALDI) mass spectrometry and proton NMR spectroscopy (^1^H NMR): 94% for PPO macromonomers and >99% for PEO macromonomers. Functionalized PPO was purified by two precipitations in cold diethyl ether. Functionalized PEO was purified by vacuum drying at 40 ºC for 7 d. Bottlebrush diblock polymer was synthesized via ring-opening metathesis polymerization with Grubbs third-generation catalyst. The catalyst was removed via chelation with SiliaMetS DMT; effluent was further processed with diatomaceous earth. Linear block copolymer P188, National Formulary Grade, was generously provided by the BASF Corporation (Vandalia, IL).

### FDB Muscle Fiber Isolation.

FDB skeletal muscle was harvested from each of the hindlimbs of mdx mice in a solution of Krebs-Henseleit Buffer Modified (K3753, Sigma-Aldrich) supplemented with 2,3-butanedione monoxime (B0753, Sigma-Aldrich) and sodium bicarbonate (S5761, Sigma-Aldrich). Following dissection, the FDB muscle was enzymatically digested in Clear M199 medium (M3769, Sigma-Aldrich; pH 7.4) also containing 0.2 g/L bovine serum albumin (A9647, Sigma-Aldrich), 4.6 mM HEPES (BP310-1, ThermoFisher Scientific), 10% fetal bovine serum (S11550, R&D Systems), and 0.2% collagenase type II (LS004176, Worthington Biochemical Corporation) at 37 °C for three hours. FDB myofibers were then triturated using a Pasteur pipette and plated onto a coverslip with 20 μg/mL laminin (23017015, ThermoFisher Scientific). After the initial myofiber adhering period, the myofibers were incubated until experimentation at 37 °C and 5% CO_2_ in a solution of Red M199 (31100-035, Life Technology; pH 7.4), 26.1 mM sodium bicarbonate, 22.9 mM HEPES, 0.2 g/L bovine serum albumin, 1x U/mL penicillin-streptomycin (1514-122, ThermoFisher Scientific), and 1 × ITS media supplement (I1884, Sigma-Aldrich; 5 mg/L insulin, 5 mg/L transferrin, 5 μg/L sodium selenite). All FDB myofibers were used within 10 h of the initial harvest.

### Sarcomere Length Dynamics.

A Nikon Eclipse TE2000-U microscope, along with the MyoCam-S and Myocyte Contractility Recording System (IonOptix), was used to measure sarcomere dynamics at 40× magnification. The field stimulation frequency was 0.2 Hz and stimulation set at 25 V. FDB myofibers were bathed with Clear M199 medium and the initial contractility was recorded. BB polymer, diluted in Clear M199 medium, was then applied. Following a 10-min incubation period, FDB myofiber contractility was retested. The evaluation of the same FDB myofiber pre- and post–polymer treatment increased the statistical power of the experiment. All FDB myofibers tested were tracked pretreatment and located posttreatment using the Micro-Manager 1.4 experimental platform. Contractions for each fiber were recorded for a minimum of 30 s after which contractions were analyzed and averaged. FDB myofibers were kept at 25 °C throughout the data collection process.

### In Vivo Mdx Studies.

The BB polymer was dissolved in sterile saline to an initial working stock solution of 50 mg/mL. BB polymer was then dissolved in sterile, filtered saline to a final working solution of 0.477 μM in sterile glass blood tubes. *Isoproterenol solution*. Isoproterenol hydrochloride (Iso; Sigma #I6504) was dissolved in saline and sterile filtered into a foil-wrapped red-top glass vial prior to injection. The sterile isoproterenol solution was stored at 4 °C for no more than 3 d, and any solution developing discoloration, indicative of degradation, was discarded.

#### BB polymer administration in postnatal mdx mice.

Starting postnatal day 1 or 2, the pups received a subcutaneous injection of the BB polymer at 0.15 mg/kg every four days (see timeline in Fig. 3) up to day 21 (P1/2, P5, P9, P13, P17, and P21). Ensuring gentle and nondisturbing handling of the mice pups is essential to avoid any potential rejection of the litter by the dam. No tail grabbing was used during the first two weeks to minimize handling stress. On postnatal day 24, mice were euthanized using phenobarbital. Blood was collected from the submandibular vein, clotted on ice, and centrifuged for serum isolation. Freshly excised skeletal muscles, tibialis anterior and diaphragm muscles, were rinsed in ice-cold PBS, dried, embedded in Tissue-Plus Optimal Cutting Temperature (OCT) Compound (Fisher Healthcare), frozen in liquid nitrogen-cooled isopentane, and stored at −80 °C for histological processing.

#### Creatine kinase MM quantification.

Serum creatine kinase levels were measured using a colorimetric creatine kinase MM ELISA kit (Novus Biologicals NBP2-75306) following the manufacturer’s instructions. After sample incubation in the CK micro-ELISA Plate, a biotinylated detection antibody was added, followed by the horseradish peroxidase HRP conjugate. After incubations and washing, the substrate reagent was added followed by the stop solution. The HRP signal was measured within 5 min via plate reader at 450 nm absorbance. Values are proportional to the amount of analyte bound to the micro-ELISA plate. Samples were run in duplicate, and values were calculated from fitted standard curves.

#### Kidney/Liver enzymes.

Serum samples were submitted to the Clinical Pathology Laboratory, University of Minnesota Veterinary Medical Center for biochemistry analysis. Creatine, blood urea nitrogen (BUN); alkaline phosphatase (ALP); aspartate transaminase (AST); and alanine transaminase (ALT) were measured.

#### Histology and staining.

Heart ventricles, diaphragm, and skeletal muscles were dissected and placed in OCT and frozen in liquid nitrogen-cooled isopentane. Tissues were sectioned transversely (seven microns) using the Leica cryostat at –20 °C. Staining was performed on unfixed tissue following standard laboratory protocols. Briefly, fresh frozen tissue sections were rehydrated in 1 × PBS, blocked with 10% normal goat serum (Jackson Immuno Research, 005-000-121), washed with PBS, and stained with the antibodies of interest. The slides were then mounted with ProLong Gold antifade mounting media with DAPI (ThermoFisher). Wheat germ agglutinin staining with DAPI was used to measure CSA and central nucleation. Immunofluorescence staining for laminin (anti-mouse laminin ThermoFisher, PA5-22901) and anti-mouse IgG 594 (Invitrogen, A11032) used to measure cardiac myocyte membrane leakiness/injury after the Iso stress test.

#### Assessment of central nucleation.

TA and diaphragm transverse sections were stained with wheat germ agglutinin (WGA) and DAPI. Images were obtained using the AXR1 Nikon confocal and Leica MICA microscope. Total and centrally nucleated fibers (CNF) were counted manually across 3,410 to 4,927 fibers, from five to seven mice per group. Results were expressed as the mean percentage of CNF per muscle.

#### CSA.

Muscle CSA was measured on wheat germ agglutinin-stained transversal sections of the tibialis anterior and diaphragm muscles using MyoVision software (41). A total of 7835 TA fibers (average 773.5 fibers per mouse) and 4030 diaphragm fibers (average 409 fibers per mouse) from five to seven mice were counted per group. Sirius red fast green staining (SRFG) was performed following standard laboratory protocol, and images were obtained using a Leica MICA microscope. The fibrotic area (red) and the total muscle area were measured using the color threshold tool in ImageJ, and fibrosis extent was calculated as the percentage of “red” area over the total tissue area.

#### Cardiac stress testing and membrane injury analysis.

Prior to the experiment, polymers were dissolved in sterile saline to final stock solution of 50 mg/mL. Bottlebrush polymer was first dissolved in deionized water to a stock solution of 300 μM and then dissolved in sterile filtered saline to a final working solution of 0.477 μM. All polymers were sterile-filtered through a PVDF membrane into a sterile glass blood tube. Isoproterenol hydrochloride (Iso; Sigma #I6504) was dissolved in saline and sterile filtered into a foil-wrapped red-top glass vial prior to injection. The sterile Iso solution was stored at 4 °C for no more than 3 d, and any solution developing discoloration, indicative of degradation, was discarded. Adult mdx mice (ages 2-5 mo) were divided into groups: 1) control, no Iso; 2) Iso + 0.9% saline; 3) Iso + P188 (460 mg/kg); and 4) Iso + bottlebrush polymer (0.15 mg/kg). WT (C57BL/10 J) mice were divided into two groups: control no Iso and Iso + 0.9% saline group. At day -1 (24 h before Iso injection) and at day 0, mice received specific dosages of polymers or equivalent saline volume subcutaneously (SubQ: beneath the scruff on the back of the neck). At day 0, mice received a single IP bolus injection of 10 mg/kg Iso in volumes of 120-150μL adjusted for body weight. At 30 h after Iso injection, blood was collected and hearts were harvested and crosscut in the middle of ventricles. The base of the heart was quickly frozen in liquid nitrogen for protein and genetic analyses, and the apex portion was embedded in Tissue-Plus OCT Compound (Fisher Healthcare) without fixation and snap-frozen for histological staining.

#### Cardiac histopathology.

For IgG staining, freshly frozen tissue sections (7 μm) were rehydrated in 1 × PBS, blocked with 10% normal goat serum (Jackson Immuno Research, 005-000-121), washed with PBS and stained with 1:100 Laminin conjugate DyLight 488 (ThermoFisher, PA5-22901) overnight at 4 °C. Slides were then washed with PBS and incubated with 1:200 goat anti-mouse IgG 594 (Invitrogen, A11032) for 1 h at room temperature. The slides were mounted with ProLong Gold antifade mountant with DAPI (ThermoFisher). The automatically montaged images of whole heart sections were acquired at 4× magnification on a Keyence BZ-X series microscope (KEYENCE) using BZ-X800 Analyzer software. The IgG-positive area was calculated using ImageJ (percentage = the number of pixels in the thresholded IgG channel/the number of pixels of myocardial tissue × 100).

#### Actimeter live animal recordings.

Control, C57BL/10ScSn, and mdx adult mice (ages 3 to 9 mo) were transported to a humidity/temperature-controlled environment and individually caged and left to acclimatize to the new environment for five days. Animals were fed a standard diet with full access to water. The Actimeter infrared laser system was aligned over each individual cage to monitor activity by recording beam break counts every 2 min. Movement counts were collected from each mouse for three days postacclimation. The mdx mice were then stratified into treatment or control groups based on the average counts derived from the individual count sum of each mouse to assure even distribution of daily activity counts per group. Mice in the treatment group were pretreated with one subcutaneous injection of BB polymer at 0.15 mg/kg, while those in the control group received equal volume of saline. One intraperitoneal injection of isoproterenol hydrochloride at 10 mg/kg and an additional injection of BB or saline was administered to each mouse the following morning. Movement counts were then collected and observed for four days prior to euthanasia according to the approved procedure by the University of Minnesota IACUC.

#### Modeling and Molecular Dynamics Simulation.

The 3-dimensional structure of the BB polymer was initially generated in 1D SMILES (Simplified-Input Line-Entry System, .smi) format (42), followed by energy minimization using OPLS-AA 2005 force field (43) with the Generalized Born implicit solvent model (44). The partial charge distribution (δ) for PEO and PPO polymer atoms was optimized based on prior quantum chemical calculations (15). The BB polymer structure was solvated in an orthorhombic system using the TIP3P explicit solvent model, with a 10 Å buffer and electroneutralized to 0.15 M via Na^+^ and Cl^–^ counterions. All-atom molecular dynamics (MD) simulations were performed using Desmond, at 310 K, 1 atm pressure, periodic boundary conditions applied via particle mesh Ewald (PME), and SHAKE to restrain hydrogen bonds (45). The initial BB polymer model was equilibrated with a 150 ns simulation under NPT ensemble conditions. To interrogate polymer–bilayer interaction, the prepared polymer was then embedded into a POPC (1-palmitoyl-2-oleoyl-glycero-3-phosphocholine) lipid bilayer in three different orientations: A) PEO side chains inserted and backbone perpendicular to bilayer X/Y plane, B) PEO side chains inserted with backbone parallel to bilayer X/Y plane, or C) PPO side chains inserted with backbone perpendicular to bilayer X/Y plane (*SI Appendix*, Fig. S4). For each orientation, a series of MD simulations were performed in triplicate using different random seeds. Each production simulation was carried out with a default initialization protocol, followed by 10 ns NPT (isothermal–isobaric) and 25 ns NPAT (constant lateral surface area) to allow lipids to relax into their preferred density around the polymer. To interrogate BB polymer–bilayer interaction under elevated stress, a series of 20 ns NPγT simulations were performed with constant lateral surface tension (γ) incremented by 1,000 bar Å at each step until structural failure of the bilayer. Post-MD analyses were performed using Maestro, VMD, and GraphPad Prism. To quantify the position of polymers relative to the bilayer during neutral stress simulations (NPT, NPAT), the minimum distance between each polymer (central backbone carbon atom 1392) and bilayer lipids (POPC nitrogen or phosphorus atoms) was calculated over time from the equation

[1] $$dmin(t)=min[d_{P,L}(t)],$$

where *d*_min_(t) is the minimum distance between the polymer (P) and lipids (L) at time t. The difference between the initial polymer–lipid distance *d*_min_(0) and the minimum distance at each timepoint *d*_min_(t) was taken to determine the change in polymer distance relative to the bilayer over time (∆*d*_min_).

## Supplementary Material

Appendix 01 (PDF)

## Data Availability

All study data are included in the article and/or *SI Appendix*.
