# Supplementary material for: Synthetic bottlebrush block copolymer prevents disease onset in Duchenne muscular dystrophy
Source: Proc Natl Acad Sci U S A. 2025 Oct 13;122(42):e2513599122. doi: 10.1073/pnas.2513599122 (PMC12557544; doi:10.1073/pnas.2513599122)
Supplement: Supplementary file 1 — Appendix 01 (PDF) [file pnas.2513599122.sapp.pdf]

PNAS

Supporting Information for

Synthetic Bottlebrush Block Copolymer Prevents Disease Onset in Duchenne Muscular Dystrophy

Houda Cohen[1], Addeli Bez Batti Angulski[1], Joseph D. Quick[1], Taylor Kuebler[1,4], Brian R. Thompson[1], John Bauer[1], Dongwoo Hahn[1], DeWayne Townsend[1], Joseph F. Hassler[2], Benjamin J. Hackel[2], Timothy P. Lodge[2,3], Yuk Y. Sham[1,4], Frank S. Bates[2], Joseph M. Metzger[1+]

+Corresponding author:
Joseph M. Metzger, Ph.D.
Email: metzgerj@umn.edu

This PDF file includes:

Figures S1 to S4

Table S1

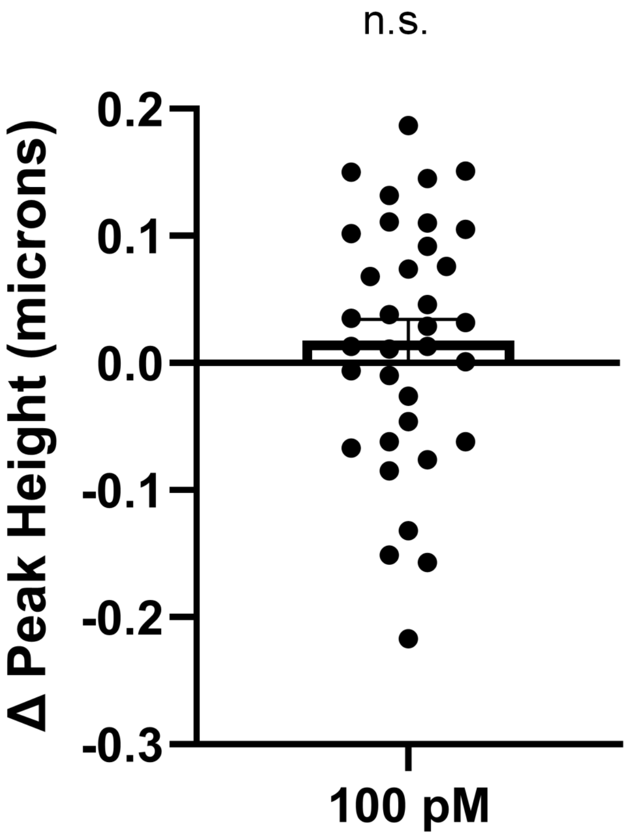

Supplemental Figure 1. Summary of effects of 100 pM bottlebrush polymer on mdx FDB fiber SL shortening amplitude during twitch contractions. The change in peak height from 100 pM BB minus pretreatment for every myofiber with mean ± SEM, not statistically significant (n.s.) with one sample t-test from zero. N=35 pairs of FDB fibers.

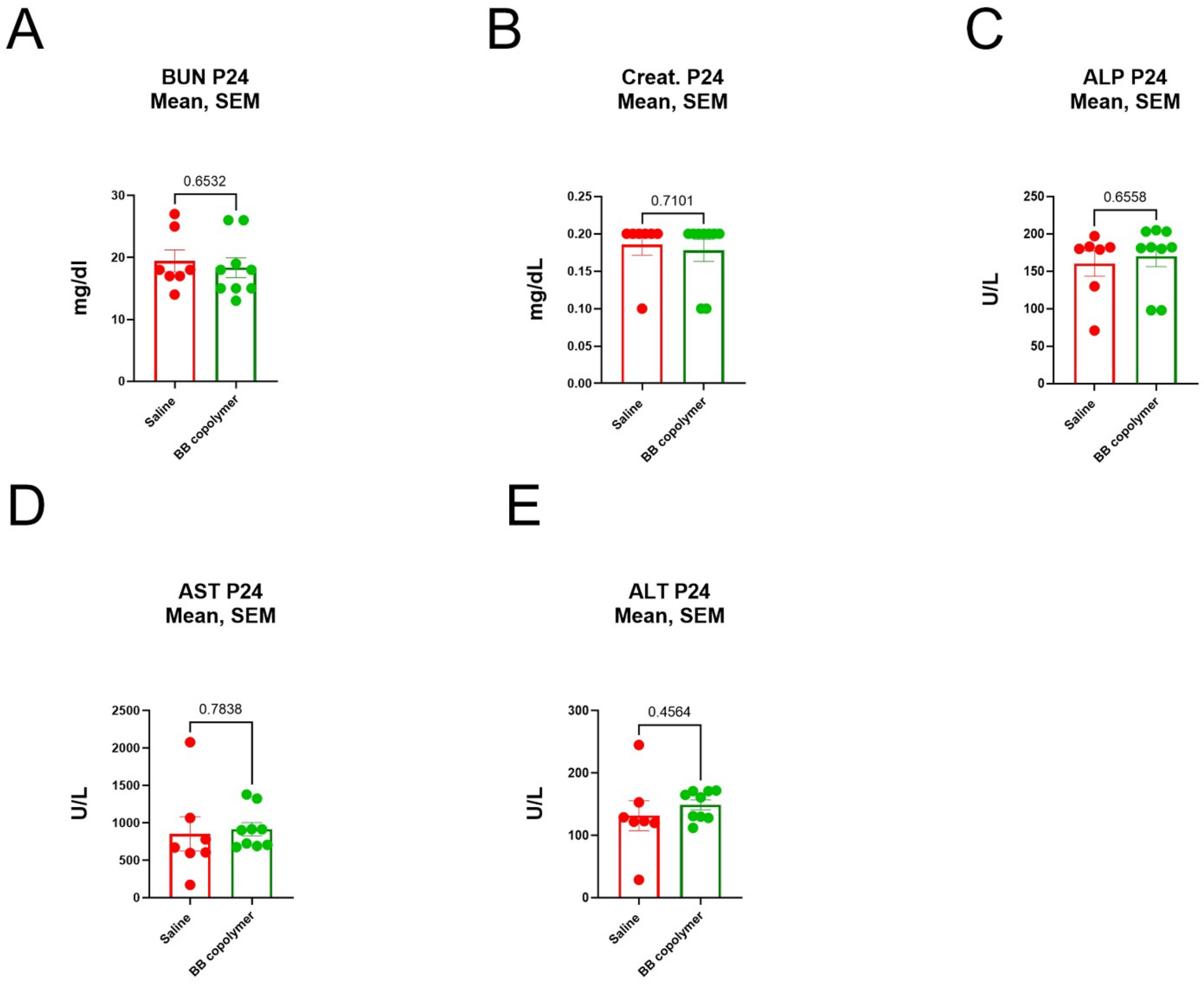

Supplemental Figure 2. Summary of serum clinical chemistry profiles in mdx mice following bottlebrush polymer treatment. Treatment protocol is as show in Figure 3A. No significant differences were obtained between saline and bottlebrush polymer groups for BUN, ALP, AST, or ALT. Data are mean ± SEM, N = 7-9 mice per group.

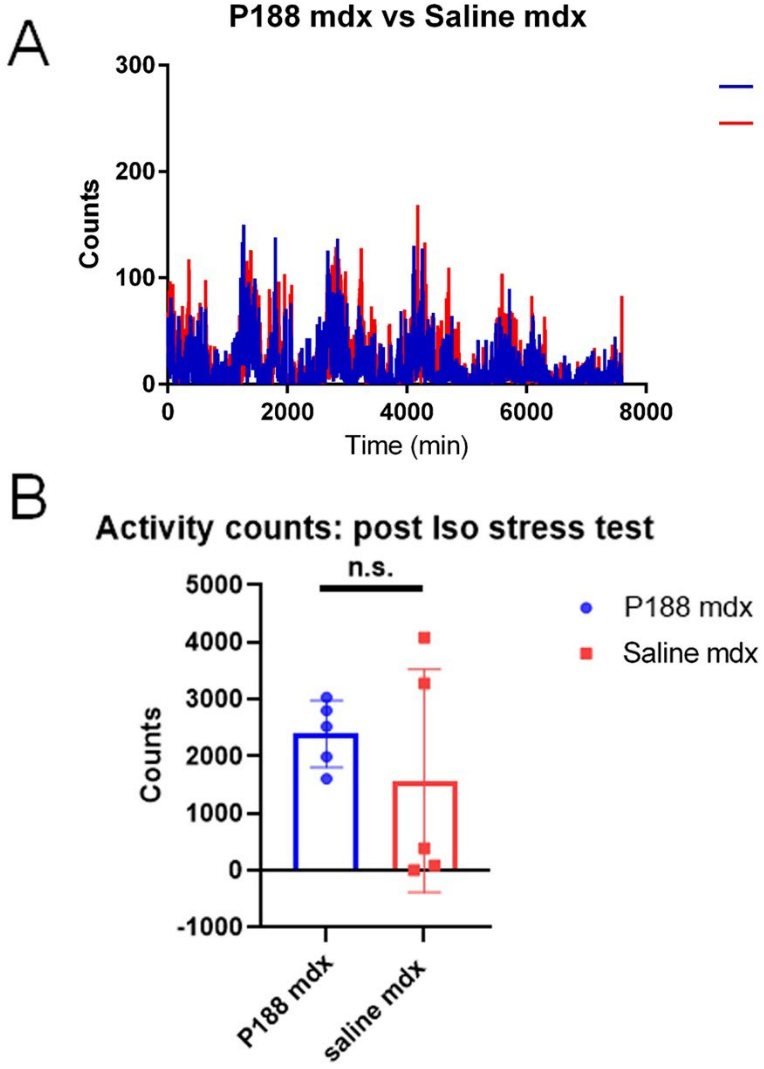

Supplemental Figure 3. Animal stress testing in unrestrained dystrophin-deficient mice *in vivo*. Mdx mice are tested as detailed in the protocol in figure 5A. A. Ensemble activity records. Time point reference: P188/saline pretreatment- 4316 min; Iso (1) - 5736 min; Iso (2) - 5890 min;
Iso (3) -6092 min. B. Summary count data, post Iso injections. P188 (460 mg/kg) treatment was not significantly different (n.s.) from saline control group in terms of animal activity counts post Iso stress testing. Data are mean ± SEM, N = 5 mice per group.

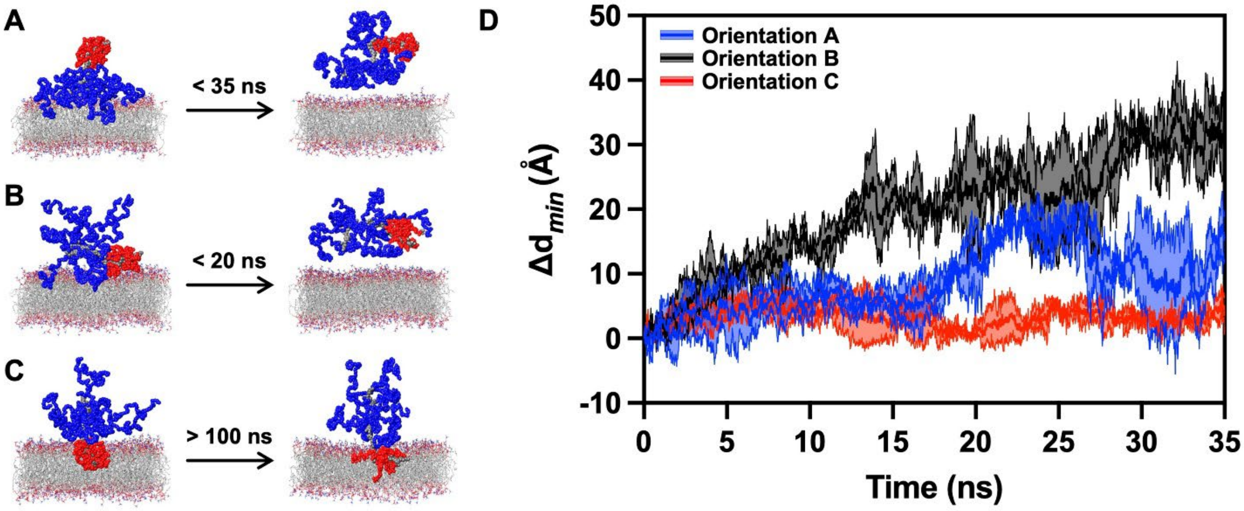

Supplemental Figure 4. All atom molecular dynamics simulations of bottlebrush polymer with POPC lipid bilayer. (A-C) Neutral tension (NPT, NPAT) simulations were performed with polymer (PEO, blue; PPO, red) embedded within a POPC bilayer in three orientations. (D) Relative change in minimum polymer-bilayer distance over time averaged across triplicates for each embed orientation (orientation A, blue; orientation B, black; orientation C, red). Here, the position of the BB polymer relative to the bilayer is plotted as the minimum distance between BB polymer central backbone carbon atom 1392 and bilayer lipids (POPC nitrogen or phosphorus atoms) calculated over time.

**Table I**	C57BL/10	Mdx	Mdx
	Saline	Saline	P188
Mean	1.094	13.03	4.963
SEM	0.3324	4.039	1.627
N	11	11	11

Cardiac stress test. IgG accumulation in myocytes post Iso (Fig. 3). One-way ANOVA, Dunnett's multiple comparisons test: mdx saline versus mdx P188, n.s.; P>0.05.
